# Biosorptive removal of acid orange 74 dye by HCl-pretreated *Lemna* sp.

**DOI:** 10.1371/journal.pone.0228595

**Published:** 2020-02-06

**Authors:** Jessica Lizeth Reyes-Ledezma, Daniel Uribe-Ramírez, Eliseo Cristiani-Urbina, Liliana Morales-Barrera

**Affiliations:** Departamento de Ingeniería Bioquímica, Unidad Profesional Adolfo López Mateos, Instituto Politécnico Nacional, Escuela Nacional de Ciencias Biológicas, Mexico City, Mexico; Qatar University, QATAR

## Abstract

Acid orange 74 (AO74) is a chromium-complex monoazo acid dye widely used in the textile industry. Due to being highly toxic and non-biodegradable, it must be removed from polluted water to protect the health of people and the environment. The aim of this study was two-fold: to evaluate the biosorption of AO74 from an aqueous solution by utilizing HCl-pretreated *Lemna* sp. (HPL), and to examine dye desorption from the plant material. The maximum capacity of AO74 biosorption (64.24 mg g^-1^) was reached after 4 h at the most adequate pH, which was 2. The biosorption capacity decreased 25% (to 48.18 mg g^-1^) during the second biosorption/desorption cycle and remained essentially unchanged during the third cycle. The pseudo-second-order kinetics model concurred well with the experimental results of assays involving various levels of pH in the eluent solution and distinct initial concentrations of AO74. NaOH (0.01 M) was the best eluent solution. The Toth isotherm model best described AO74 biosorption equilibrium data. FTIR analysis confirmed the crucial role of HPL proteins in AO74 biosorption. SEM-EDX and CLSM techniques verified the effective biosorption/desorption of the dye during the three cycles. Therefore, HPL has potential for the removal of AO74 dye from wastewaters.

## Introduction

Azo dyes are aromatic compounds that constitute the largest and the most important group of synthetic dyes [1, 2]. Metal-complex azo acid dyes are widely used in the textile industry due to their light and wet fastness properties [3]. They have an affinity to protein fibers and polyamide but not to cellulosic fibers. Their structure includes solubilizing groups (e.g., hydroxyl, carboxyl and amino) [4] capable of forming coordination complexes with metal ions, mainly chromium, copper, cobalt, iron and nickel. The metal-complex dyes most frequently found in industry are those containing chromium [1].

Acid orange 74 (AO74), a chromium-complex monoazo acid dye, is an efficient resource of industry employed mainly for coloring wool, silk, nylon, leather and polyamide fiber [1]. Its chromium ion bound to the azo group provides great stability to the dye structure during its photocatalytic degradation [5]. Because the enzymatic discoloration of AO74 rarely occurs, it is classified as a non-biodegradable dye [6, 7].

Given the toxic, mutagenic and carcinogenic properties of metal-complex azo dyes [8], their elevated concentration in wastewater represents a grave threat to public health and aquatic life. Aromatic amines produced by dye degradation possess the same harmful properties [2, 9]. Thus, it is essential to remove these dyes from wastewater to prevent them from being released into the environment. Treatment systems for dye-containing wastewater are mainly based on physicochemical methods, such as activated carbon sorption, chemical precipitation and degradation (ozonation and Fenton reactions), photodegradation, flocculation, reverse osmosis, ion exchange [10] and catalytic wet air oxidation [11]. Biological methods generally consist of dye decolorization processes [12], which are either expensive, non-specific, or generate more toxic compounds than the original dye [13].

Biosorption is an economical alternative for wastewater treatment. It uses low-cost natural materials that are highly efficient at removing toxic compounds and have an acceptable environmental impact. The biosorption of anionic dyes has been evaluated in studies on water-insoluble biopolymers such as chitosan and cellulose. Chitosan has shown stability and a great sorption capacity in acidic media [14,15]. On the other hand, cellulose (the most abundant polymer in nature) is found in agro-industrial wastes and with a minimum of processing can be utilized for the removal of cationic and anionic dyes [16, 17].

The biosorbent presently under investigation is *Lemna* sp., a plant rich in cellulose. Commonly known as duckweed, this macrophyte proliferates rapidly and adapts to a wide range of pH and temperatures in lakes, ponds, canals and slow-moving streams, and therefore is often considered a nuisance [18, 19]. Despite the extensive research on *Lemna* sp. as a heavy-metal biosorbent [20, 21, 22, 23, 24, 25, 26], scarce reports address its capacity to remove dyes from aqueous solutions [27, 28].

The aim of the current study was to evaluate HCl-pretreated *Lemna* sp. (HPL) as a biosorbent of AO74 in water, including the examination of its efficiency during various biosorption/desorption cycles. Fourier-transform infrared (FTIR) spectroscopy, scanning electron microscopy with energy dispersive X-ray (SEM/EDX) spectroscopy, and confocal laser scanning microscopy (CLSM) revealed the presence of the AO74 dye on the surface of *Lemna* sp. and allowed for the identification of the biosorption sites on the plant material.

## Material and methods

### Ethics statement

Local regulations do not require any special permits for the collection of *Lemna* sp. samples. The studies conducted in this work did not involve any endangered or protected species.

### Chemicals

**Fig 1:**
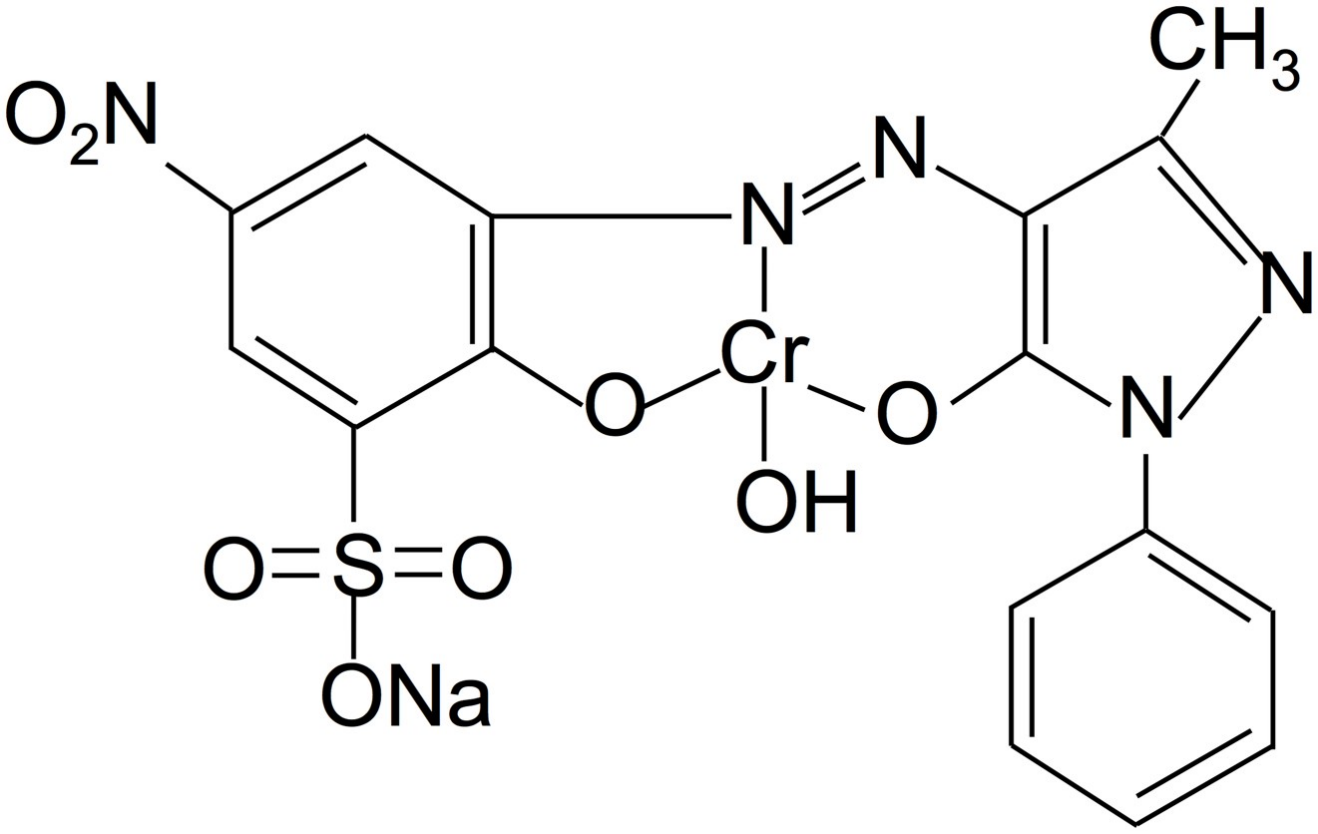
Molecular structure of AO74.

The molecular structure and principle characteristics of AO74 are shown in Fig 1 and Table 1, respectively. AO74 dye was purchased from Sigma-Aldrich (>90% purity). All other reagents were of analytical grade. The AO74 stock solution was prepared in deionized water (1 g L^-1^) and diluted to prepare the test solutions.

**Table 1:** Principle characteristics of AO74.

Name	Acid orange 74
C.I.	18745
CAS No.	10127-27-2
Chemical formula	C_16_H_11_CrN_5_NaO_8_S
Molecular weight	508.34 g mol^-1^
λ_max_	478 nm
Type	Chromium-complex monoazo acid dye (anionic dye)

### Biosorbent

*Lemna* sp. was harvested in San Gregorio Atlapulco, Xochimilco, Mexico City (19.2588422, -99.0848157). After exhaustive washing, first with abundant fresh water and then with distilled deionized water, the plant was dried in an oven at 60 °C for 48 h. Subsequently, it was ground in a Glen Creston mill and the powder was sifted through ASTM standard sieves to obtain 0.3 to 0.5 mm particles, which were rinsed in 0.05 M HCl for 4 h under continuous agitation in an All Sheng orbital shaker at 140 rpm. The resulting HPL was washed several times with distilled deionized water (until the wash water reached neutral pH) before being dried at 60 °C for 48 h. By applying techniques detailed in the AOAC handbook [29, 30], proximate chemical analysis was performed to characterize HPL.

An evaluation of the zeta potential of HPL suspensions (1 g L^-1^) was carried out at room temperature (rt) and at different pH values (1.0–11). Such suspensions were examined in a Zeta Plus ZA to determine the net surface charge in each case.

### Biosorption studies

The kinetics of biosorption was assessed, exploring the impact of pH by running assays with distinct values of this parameter (1.5–5). Briefly, 120 mL of AO74 dye solution at 30 mg L^-1^ was placed in a 500 mL Erlenmeyer flask and the biosorbent was added at a concentration of 1 g (dry weight) L^-1^. Subsequently, the flask was agitated in an All Sheng^™^ orbital shaker (140 rpm) at rt for 24 h. The pH was maintained at a constant level throughout each assay by adding a 0.1 M HCl or NaOH solution. Samples were collected at different times and centrifuged (Sol-Bat^™^ Centrifuge C-600) at 3000 rpm for 5 min. The supernatant was appropriately diluted in distilled water to quantify the residual dye concentration in a UV/VIS spectrophotometer (Genesys^™^ 10 UV/Visible, Thermo Electron Scientific Instruments Corporation) at 478 nm. The best pH for biosorption was 2.0. Another important parameter considered was the effect of the initial AO74 dye concentration on biosorption. Hence, the kinetics of biosorption was observed using various dye concentrations (15–300 mg L^-1^) with solutions at a pH of 2.0, while maintaining other experimental conditions similar to those previously described.

The time-dependent biosorption capacity of AO74 was determined as follows:

$$q_{tB}=\frac{C_{0}-C_{res}}{M}$$ (1)

Where *C*_*0*_ is the initial dye concentration (mg L^-1^) at *t = 0* h, *C*_*res*_ is the residual concentration of the dye at any specific time *t* (mg L^-1^) and *M* is the biosorbent concentration (g L^-1^). At equilibrium, *C*_*res*_ = *C*_*e*_ and *q*_*tB*_ = *q*_*eB*_.

Theoretical biosorption capacities were analyzed with assorted kinetics models (Table 2). Biosorbent-free controls were run simultaneously with the dye biosorption evaluations, utilizing exactly the same settings. All assays were conducted in triplicate.

**Table 2:** Kinetic models of biosorption/desorption.

Model	Equation	Parameters
Pseudo-first-order	$q_{t}=q_{e}(1-e^{k_{1}t})$	*k*_*1*_—pseudo-first-order rate constant [h^-1^]
Pseudo-second-order	$q_{t}=\frac{t}{\frac{1}{k_{2}q_{e}}+\frac{t}{q_{e}}}$	*k*_*2*_—pseudo-second-order rate constant [g mg^-1^ h^-1^]
Elovich	$q_{t}=\frac{1}{B}\text{ln}\left(AB\right)+\frac{1}{B}\text{ln}\;t$	*A*—initial biosorption/desorption rate constant [mg g^-1^ h^-1^]
		*B*—constant related to the extent of surface coverage and the activation energy [g mg^-1^]
Fractional power	*q*_*t*_ = *k*_*p*_*t*^*v*^	*k*_*p*_—constant of the model [mg g^-1^]
		*v*—rate constant [h^-1^]

### Desorption studies

In order to select the best eluent solution for AO74 desorption, a sample of AO74-loaded HPL was prepared by mixing 1 g L^-1^ of HPL with a concentrated solution of AO74 dye (300 mg L^-1^) at pH 2.0. Subsequently, the mixture was shaken at 140 rpm for 24 h at rt, then centrifuged at 3500 rpm for 15 minutes to separate the AO74-loaded biosorbent, which was washed with distilled deionized water and centrifuged (maintaining the conditions previously described). This procedure was repeated two more times to remove any excess dye. The saturated biosorbent was finally dried at 60 °C for 48 h and stored to await desorption testing.

Desorption assays were initially performed with distinct eluent solutions: water (at rt, 18 °C), NaOH (0.1 M), Na_2_CO_3_ (0.1 M), NaHCO_3_ (0.1 M), NH_4_NO_3_ (0.1 M), NaCl (0.1 M), (NH_4_)_2_SO_4_ (0.1 M), NaH_2_PO_4_ (0.1 M), HCl (0.1 M) and acetone (1% v/v). To establish the best solution for desorption, 1 g L^-1^ of AO74-loaded HPL was placed in each of these eluents and the corresponding mixture was stirred constantly at 140 rpm for 3 h at rt. Upon completion of the time, the different suspensions were centrifuged and the supernatants were properly diluted to obtain the eluted dye concentration spectrophotometrically at 478 nm. Desorption was quantified as a percentage by applying the following formula:

$$\%\;Des=\frac{C_{D}-C_{0}}{q_{e}\;X}\;x\;100$$ (2)

Where % *Des* is the percentage of dye desorption after 3 h, *C*_*D*_ is the eluted dye concentration at the same time point (mg L^-1^), *C*_*0*_ is the initial dye concentration in the solution (mg L^-1^), *q*_*e*_ is the maximum amount of dye contained in AO74-loaded HPL (equivalent to the previously determined equilibrium biosorption capacity in mg g^-1^), and *X* is the concentration of dye-loaded HPL (g L^-1^).

After identifying which eluent solution showed the highest percentage of AO74 desorption from dye-loaded HPL, assays were carried out to ascertain its optimal concentration. The eluent concentration with the best desorption performance was used in further studies.

### Analysis of biosorption/desorption cycles

Biosorption/desorption cycles were evaluated for biosorbent reusability. The first biosorption step involved an AO74 solution at a concentration of 200 mg L^-1^ and a pH of 2. The initial dye concentration was established spectrophotometrically. Subsequently, 1 g L^-1^ of HPL was added and the mixture was stirred constantly (140 rpm) for 6 h at rt. Meanwhile, samples were collected and centrifuged under the same conditions to monitor the residual concentration of the dye in the supernatant as well as the biosorption capacity (*q*_*tB*_). Upon completion of the six hours, all the remaining suspension was centrifuged. The saturated biosorbent was washed with distilled deionized water to remove any excess dye and then dried at 60 °C for 48 h.

AO74-loaded HPL was subjected to the first desorption process. 1 g L^-1^ of dye-loaded biosorbent (dry weight) was added to the best eluent solution and the mixture was stirred at 140 rpm for 3 h at rt. Samples were taken periodically to measure the concentration of the dye in the solution, thus allowing for the calculation of the desorption capacity (*q*_*tD*_). Finally, the resulting suspension was centrifuged and washed with distilled deionized water to recover the eluted biosorbent. This in turn was dried at 60 °C for 48 h.

The desorption capacity (*q*_*D*_) was determined with the following equation:

$$q_{tD}=\frac{C_{tD}-C_{0}}{X}$$ (3)

Where *q*_*tD*_ is the desorption capacity (mg g^-1^), *C*_*tD*_ and *C*_*0*_ are the dye concentrations (mg L^-1^) at any specific time *t* (h) or at 0 h (*t*_*0*_), and *X* is the concentration of dye-loaded HPL (g L^-1^).

The procedure was repeated three times to scrutinize the samples for any possible change in biosorption and/or desorption capacity. A portion of the saturated and desorbed biosorbent from each cycle was retained for the FTIR and SEM/EDX studies.

### Biosorption and desorption kinetic modeling

The kinetics of biosorption and desorption of AO74 by HPL was examined with different theoretical models (Table 2).

Where *q*_*t*_ is the capacity of biosorption (*q*_*tB*_)/desorption (*q*_*tD*_) at time *t* (h), and *q*_*e*_ is the predicted capacity of equilibrium biosorption (*q*_*eB*_)/desorption (*q*_*eD*_) given by the model.

The correlation coefficient (R^2^), the sum of squared error (SSE), and the root mean standard error (RMSE) were calculated in order to select the most suitable model [31]. A comparison was then made between the experimental data and theoretical values.

### Fourier-transform infrared (FTIR) spectroscopy

FTIR spectra were recorded for AO74-unloaded and AO74-loaded HPL to evaluate changes in the functional groups of the biosorbent caused by the presence of the dye, and to identify possible functional groups involved in biosorption. Accordingly, AO74-unloaded and AO74-loaded HPL were dried in an oven at 105 °C until a constant weight was reached, thus assuring the removal of all retained water that might interfere with the detection of functional groups on the surface of the biosorbent. Each of these biosorbents (loaded and unloaded) were finely ground and mixed with spectroscopic grade KBr powder at a ratio of 1:5. Samples were analyzed by using FTIR spectroscopy (Perkin-Elmer 2000 equipped with a diffuse reflectance accessory, DRA-FTIR) in the range of 4000–400 cm^-1^ with 16 scans at a resolution of 4 cm^-1^.

### Scanning electron microscopy/energy dispersive X-Ray spectroscopy (SEM/EDX)

SEM/EDX analysis was employed to examine modifications in the structure of the HPL surface resulting from the biosorption and subsequent desorption of AO74. Samples of AO74-unloaded and AO74-loaded HPL were dried for 24 h, then covered with coal to be observed under a high-resolution scanning electron microscope (HR-SEM JEOL model JSM7800F) at an accelerating voltage of 15 kV.

### Confocal laser scanning microscopy (CLSM)

Confocal laser scanning microscopy (CLSM) was used to verify the biosorption of AO74 dye by HPL as well as the desorption of the dye from this plant material. For this study, HPL, AO74 and AO74-loaded HPL and AO74 unloaded HPL were dried at 60 °C before carrying out CLSM on an LSM-710 NLO microscope (Carl Zeiss, Germany) with a fluorescence detection range of 417–729 nm. The samples were mounted directly on glass slides. The autofluorescence signal measurements of the samples were made on the ZEN software of an LSM 710 confocal microscope. Scanning was performed with the EC Plan-Neofuar 10x/0.3 objective.

### Statistical analysis

Data are expressed as the mean ± SEM. All calculations were made on GraphPad Prism^®^ software version 7.0. Significant differences were determined by analysis of variance (ANOVA with Tukey’s test and an overall confidence level α = 0.05). All theoretical values of the kinetic models of biosorption and desorption were compared to the experimental data by non-linear regression analysis.

## Results and discussion

The present results evidence the removal of AO74 dye by HPL and not by abiotic factors.

### Biosorbent characterization

#### Proximate chemical analysis

The constituents of HPL were established by proximate chemical analysis (Table 3). *Lemna* is well-known for its high level (up to 50%) of carbohydrates (raw fiber + nitrogen free extract). The content of proteins is about 20%-30%, of minerals (as ash) 12–25% and of lipids <5%. This composition renders it ideal for use in the formulation of animal feed. Additionally, it is currently being investigated in relation to biofuel production [32, 33, 34, 35].

**Table 3:** Proximate chemical analysis of HPL.

Composition	Percentage (dry basis)
Crude Protein	25.65 ± 0.79
Ether extract (crude fat)	6.25 ± 0.20
Ash	3.05 ± 0.16
Carbohydrate	65.05 ± 0.8

Zhao et al. studied *Lemna minor* [34], identifying the main carbohydrates in the cell wall as cellulose, pectin and hemicellulose. Linoleic acid was detected in the lipid composition and phenolic compounds were found. Gusain and Suthar [35] reported an elevated ash content (approx. 18%), attributed to calcium, silicium, magnesium, sodium, potassium, sulfur and phosphorus [32, 36, 37].

The present characterization of HPL revealed a high carbohydrate and protein content, suggesting that the active biosorption sites contained in these components could have played an important role in the removal of the AO74 dye.

#### Zeta potential and point of zero charge

**Fig 2:**
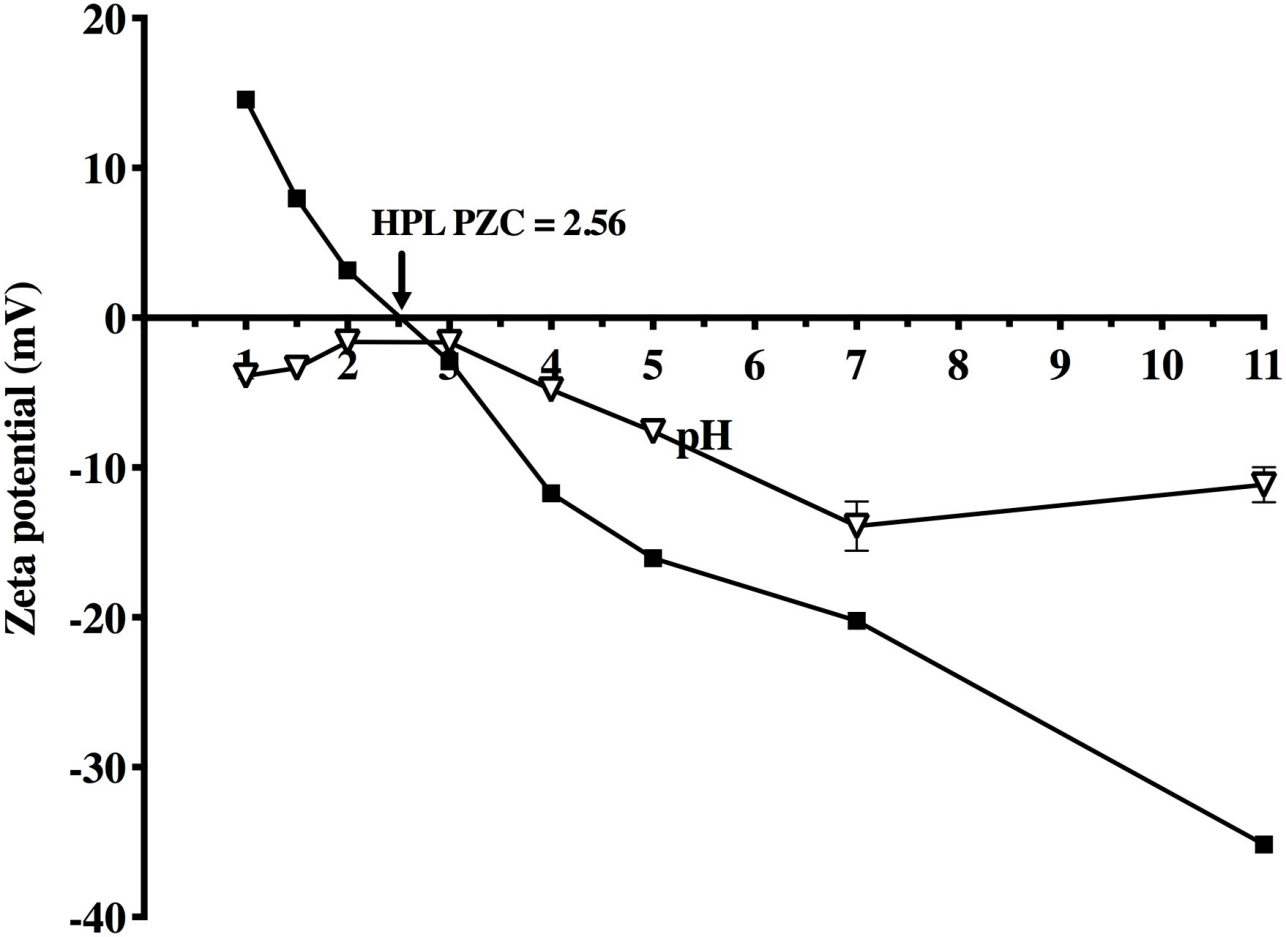
Zeta potential of HPL (■) and AO74 (▽) as a function of pH.

The zeta potential (Fig 2) of AO74 dye was negative at all levels of pH tested (due to its anionic nature), while the biosorbent surface produced a positive value for this parameter in solutions having a pH below 2.56. Therefore, the AO74 dye was drawn towards sorption sites in the solutions with pH<2.56. The point zero charge (PZC) of HPL was pH = 2.56. As the pH increased, the zeta potential decreased up to -35.16 mV because the functional groups on the HPL surface become more negatively charged at higher pH. Due to the difference in electrical charge between the dye and the biosorbent surface, electrostatic attraction is probably one of the main sorption mechanisms involved in the removal of AO74 by HPL.

According to the proximate chemical and FTIR analyses (see the following results), the two main components of HPL are carbohydrates and proteins. The hydroxyl, carboxyl, amide and amino functional groups of these components may be responsible for conferring a charge to the surface of the biosorbent.

### Biosorption studies

#### Effect of pH on the biosorption capacity of HPL for AO74

**Fig 3:**
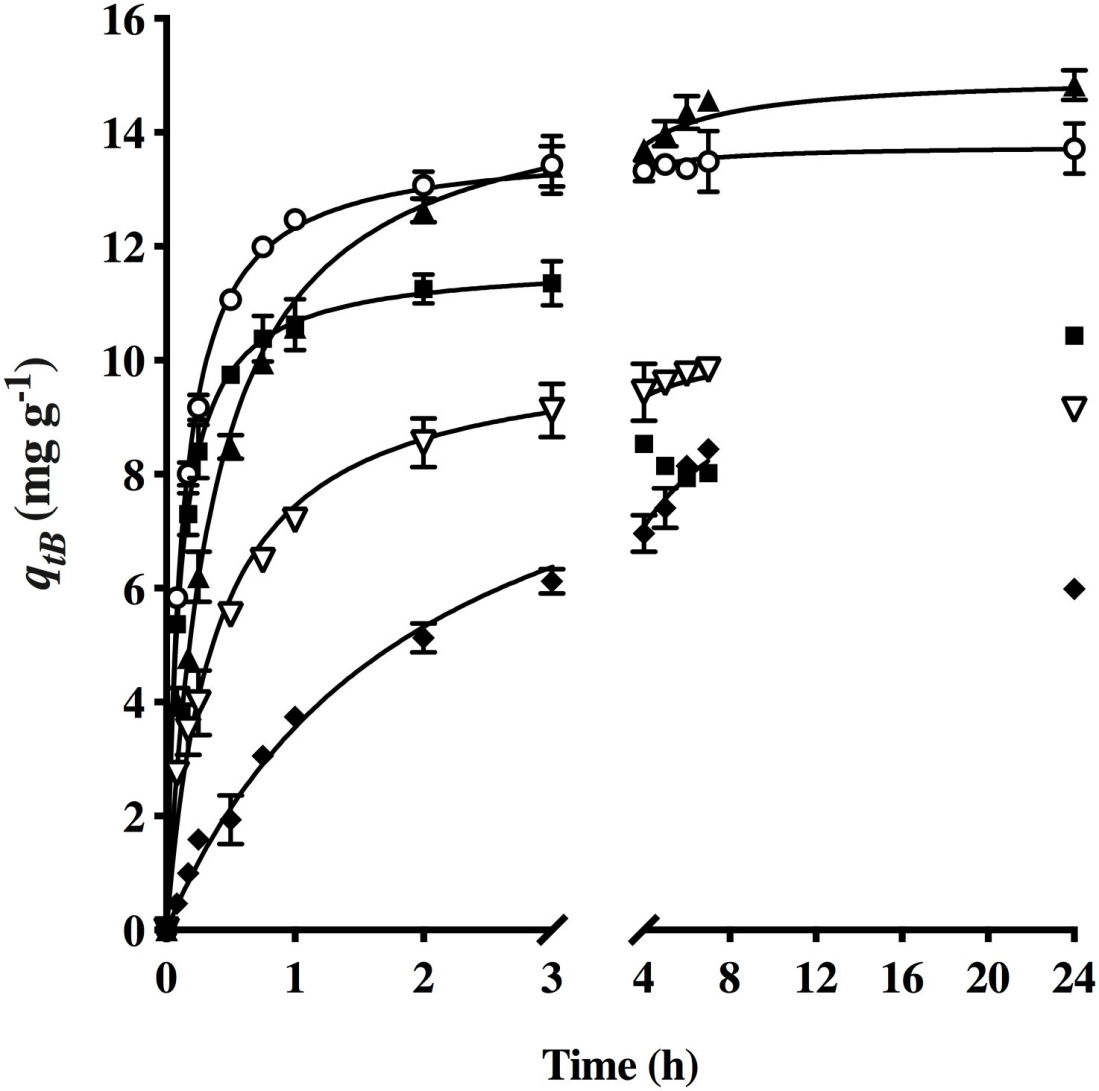
Effect of pH on the biosorption of AO74 by HPL [pH: (■) 1.5, (○) 2.0, (▲) 3.0, (▽) 4.0, (◆) 5.0] and the (—) pseudo-second-order model prediction.

The kinetics of the biosorption of HPL for AO74 was examined at different pH values. The capacity of this biosorbent was relatively low (11.08 mg g^-1^) at pH 1.5 compared to that obtained with a slight increment in pH (Fig 3). Thus, a solution with high acidity caused damage to the biosorbent structure, possibly explaining the reason for the reduction in dye removal capacity after 3 hours.

At pH 2, an equilibrium biosorption capacity of 13.51 mg g^-1^ was reached in 2 h. At pH 3 a maximum equilibrium biosorption capacity of 14.43 mg g^-1^ was achieved in 5 h. Contrarily, there was a decline in biosorption capacity at pH 4 and 5. The determination of the zeta potential revealed negatively charged biosorption sites at pH 4 and 5, which probably hindered dye biosorption due to the repulsion between the dye and the biosorbent. At the latter pH values, the decline in biosorption capacity was notable at 24 h of the assay.

Table 4 presents the experimental equilibrium biosorption capacity (*q*_*eB*_ *exp*), time required to reach equilibrium (*t*_*e*_), constants for each of the models tested (*q*_*e1*_, *k*_*1*_, *q*_*e2*_, *k*_*2*_, *A*, *B*, *k*_*p*_ and *v*) and error functions (*R*^*2*^, *SSE* and *RMSE*).

**Table 4:** Kinetic model parameters for the biosorption of AO74 dye by HPL at various levels of pH in the solution.

pH	1.5	2.0	3.0	4.0	5.0
***t***_***e***_ **(h)**	1.0	2.0	5.0	3.0	6.0
***q***_***eB***_ ***exp* (mg g**^**-1**^**)**	11.08 ± 0.23	13.51 ± 0.08	14.43 ± 0.18	9.55 ± 0.13	8.30 ± 0.15
**Pseudo-first-order model**					
***q***_***e1***_ **(mg g**^**-1**^**)**	10.74 ± 0.19	13.1 ± 0.16	13.85 ± 0.25	9.33 ± 0.20	8.21 ± 0.20
***k***_***1***_ **(h**^**-1**^**)**	6.913 ± 0.52	5.307 ± 0.34	1.953 ± 0.15	1.91 ± 0.16	0.54 ± 0.04
**R**^**2**^	0.977	0.973	0.963	0.955	0.986
**SSE**	5.006	11.25	21.87	11.18	3.214
**RMSE**	0.559	0.658	0.917	0.682	0.366
**Pseudo-second-order model**					
***q***_***e2***_ **(mg g**^**-1**^**)**	11.72 ± 0.12	13.77 ± 0.07	14.99 ± 0.15	10.23 ± 0.16	10.54 ± 0.27
***k***_***2***_ **(g mg**^**-1**^ **h**^**-1**^**)**	0.86 ± 0.052	0.618 ± 0.02	0.187 ± 0.01	0.261 ± 0.02	0.048 ± 0.01
**R**^**2**^	0.995	0.996	0.992	0.984	0.993
**SSE**	1.035	1.42	4.944	3.929	1.628
**RMSE**	0.254	0.234	0.436	0.405	0.260
**Elovich model**					
***A* (mg g**^**-1**^ **h**^**-1**^**)**	846.6± 321.2	4662 ± 3371	189.2± 40.81	90.24 ± 9.99	17.47 ± 1.52
***B* (g mg**^**-1**^**)**	0.605 ± 0.05	0.726 ± 0.07	0.442 ± 0.02	0.57 ± 0.02	0.53 ± 0.02
**R**^**2**^	0.919	0.825	0.939	0.978	0.960
**SSE**	5.18	26.86	22.22	3.485	7.47
**RMSE**	0.608	1.058	0.962	0.398	0.583
**Fractional power model**					
***K***_***p***_ **(mg g**^**-1**^**)**	10.13 ± 0.21	11.09 ± 0.27	9.729 ± 0.34	6.481 ± 0.15	3.37 ± 0.105
***v* (h**^**-1**^**)**	0.169 ± 0.02	0.109 ± 0.01	0.189 ± 0.02	0.25 ± 0.016	0.5 ± 0.02
**R**^**2**^	0.866	0.753	0.849	0.943	0.983
**SSE**	8.52	37.83	54.91	8.822	3.133
**RMSE**	0.780	1.256	1.513	0.633	0.377

The pseudo-second order equation yielded the highest correlation coefficients (*R*^*2*^) and the lowest error functions compared to the other models. Thus, this model best described the kinetics of AO74 biosorption at the different pH values. The continuous lines in Fig 3 depict the pseudo-second-order model predictions. The points at which HPL caused an evident decrease in dye concentration were not considered in the pursuit of a kinetic model. Since time is an important factor for the feasibility of a process, pH 2 was selected as optimum for the removal of AO74 by HPL, and therefore was used in further studies.

#### Effect of the initial AO74 concentration on biosorption capacity

**Fig 4:**
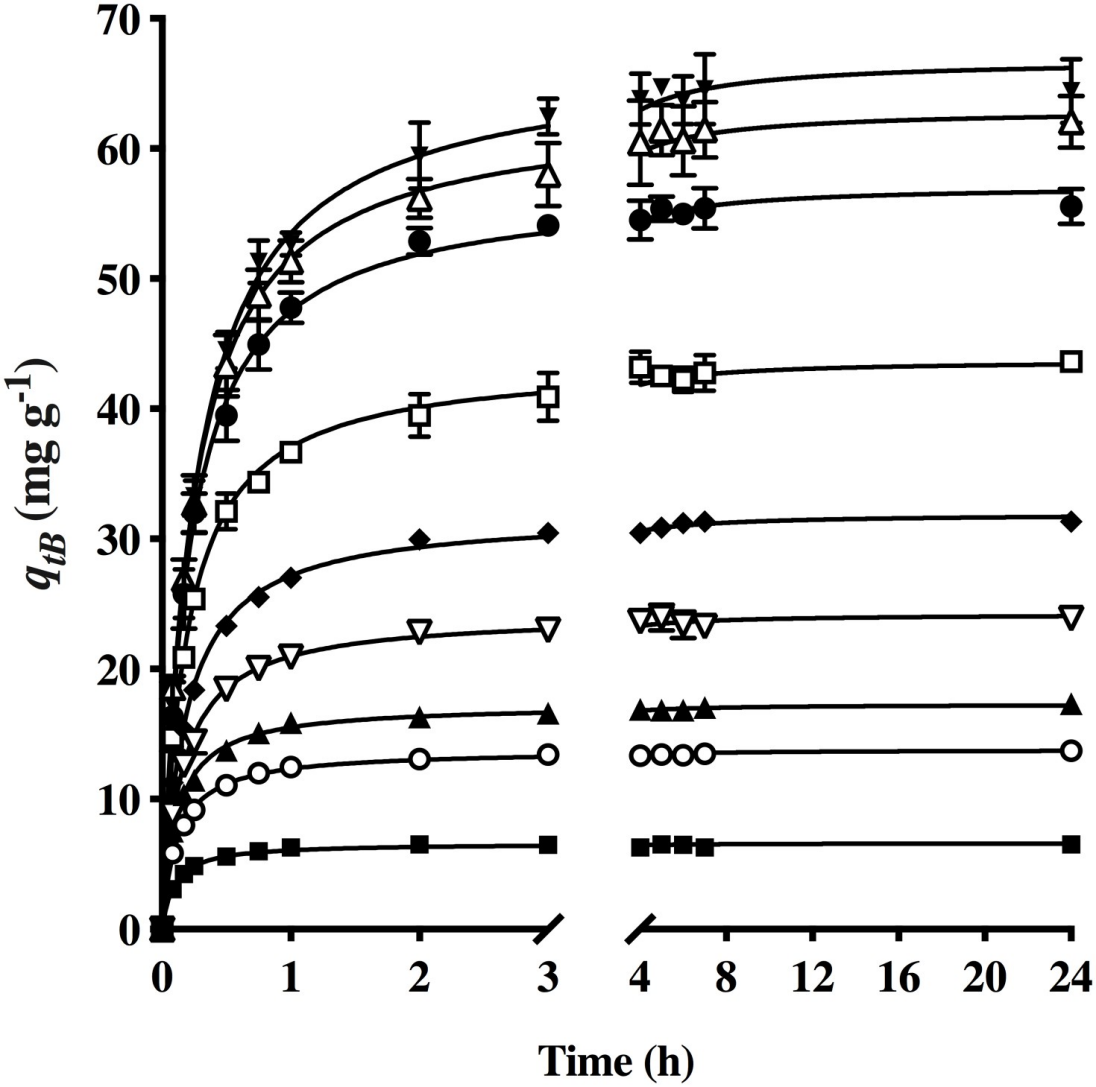
Effect of the initial AO74 concentration on dye biosorption by HPL [C_0_ (mg L^-1^): (■) 15, (○) 20, (▲) 40, (▽) 60, (◆) 80, (□) 100, (●) 150, (△) 200 and (▼) 300] and (—) the pseudo-second-order model prediction.

When testing the biosorption kinetics of AO74 at various initial concentrations (Fig 4), it was found that an increase in the initial concentration (from 15 to 300 mg L^-1^) led to a rise in the biosorption capacity of HPL (from 6.17 to 64.24 mg g^-1^).

It was previously reported that the initial dye concentration provides the driving force for overcoming the mass transfer resistance between the sorbate and the biosorbent in the solution [38]. A higher initial concentration of the sorbate enhances the probability of it colliding with the biosorbent, thus improving the biosorption capacity. In accordance with this idea, the uptake capacity was about 5% greater at 300 versus 200 mg L^-1^ of AO74. On the other hand, a small or null increment in the biosorption capacity reveals that no more sorption sites are available for dye removal and the maximum uptake value has been reached [39].

The experimentally evaluated kinetic parameters included the equilibrium removal capacity (*q*_*eB*_ *exp*) and the time required to reach equilibrium (*t*_*e*_) (Table 5).

**Table 5:** Kinetic model parameters for the biosorption of AO74 by HPL at distinct initial dye concentrations.

*C*_*0*_ (mg L^-1^)	15	30	40	60	80	100	150	200	300
***t***_***e***_ **(h)**	0.25	0.5	0.75	1.0	2.0	3.0	3.0	4.0	4.0
***q***_***eB***_ ***exp* (mg g**^**-1**^**)**	6.17 ± 0.16	12.94 ± 0.27	16.53 ± 0.23	23.11 ± 0.34	30.8 ± 0.20	42.54 ± 0.38	54.99 ± 0.24	61.19 ± 0.30	64.24 ± 0.20
**Pseudo-first-order model**									
***q***_***e1***_ **(mg g**^**-1**^**)**	6.33 ± 0.04	13.1 ± 0.09	16.44 ± 0.12	22.88 ± 0.18	30.09 ± 0.23	41.11 ± 0.36	53.71 ± 0.43	59.03 ± 0.53	62.49 ± 0.49
***k***_***1***_ **(h**^**-1**^**)**	6.561 ± 0.240	5.307 ± 0.191	5.445 ± 0.220	4.162 ± 0.164	3.71 ± 0.143	3.679 ± 0.160	3.248 ± 0.123	3.014 ± 0.127	2.71 ± 0.096
**R**^**2**^	0.972	0.973	0.965	0.968	0.969	0.961	0.971	0.946	0.975
**SSE**	7.675	33.74	66.71	130.2	220.3	525.9	693.0	1050.0	859.5
**RMSE**	0.306	0.642	0.902	1.260	1.639	2.533	2.907	3.578	3.238
**Pseudo-second-order model**									
***q***_***e2***_ **(mg g**^**-1**^**)**	6.61 ± 0.04	13.77 ± 0.04	17.28 ± 0.07	24.21 ± 0.1	31.93 ± 0.08	43.73 ± 0.20	57.16 ± 0.28	63.03 ± 0.36	66.88 ± 0.34
***k***_***2***_ **(g mg**^**-1**^ **h**^**-1**^**)**	1.685 ± 0.071	0.617 ± 0.012	0.507 ± 0.015	0.266 ± 0.007	0.179 ± 0.003	0.126 ± 0.004	0.086 ± 0.003	0.071 ± 0.003	0.059 ± 0.002
**R**^**2**^	0.986	0.997	0.993	0.993	0.998	0.992	0.992	0.989	0.992
**SSE**	3.966	4.259	13.88	28.19	17.71	102.2	202.3	3.23.5	278.3
**RMSE**	0.220	0.228	0.411	0.586	0.465	1.116	1.571	1.986	1.842
**Elovich model**									
***A* (mg g**^**-1**^ **h**^**-1**^**)**	11476 ± 7665	4662 ± 1894	6563 ± 2675	2609 ± 832.2	2258 ± 595.7	2636 ± 652.1	2440 ± 631.6	2105 ± 465.8	1650 ± 354.1
***B* (g mg**^**-1**^**)**	1.784 ± 0.126	0.726 ± 0.038	0.586 ± 0.031	0.363 ± 0.018	0.262 ± 0.012	0.187 ± 0.008	0.137 ± 0.007	0.120 ± 0.005	0.109 ± 0.005
**R**^**2**^	0.725	0.825	0.828	0.843	0.871	0.879	0.583	0.878	0.870
**SSE**	23.85	80.58	120.8	282.6	432	791.9	1841	1936	2531
**RMSE**	0.560	1.03	1.231	1.928	2.384	3.228	4.921	5.047	5.771
**Fractional power model**									
***k***_***p***_ **(mg g**^**-1**^**)**	5.532 ± 0.076	11.09 ± 0.151	13.97 ± 0.184	18.65 ± 0.296	24.07 ± 0.385	32.73 ± 0.527	41.87 ± 0.795	45.34 ± 0.845	46.98 ± 0.980
***v* (h**^**-1**^**)**	0.090 ± 0.008	0.109 ± 0.008	0.107 ± 0.007	0.126 ± 0.009	0.135 ± 0.009	0.139 ± 0.009	0.145 ± 0.010	0.153 ± 0.010	0.160 ± 0.011
**R**^**2**^	0.663	0.7531	0.7601	0.7616	0.7862	0.7959	0.7595	0.7869	0.7696
**SSE**	29.22	113.5	169	429	717.8	1335	3015	3377	4497
**RMSE**	0.620	1.222	1.491	2.376	3.073	4.191	6.299	6.666	7.692

Compared to all the other kinetic models, the pseudo-second-order equation herein rendered higher correlation coefficients (*R*^*2*^) and lower *SSE* and *RMSE* values. Consequently, it is considered the most appropriate model for describing biosorption kinetics at different initial dye concentrations. Hence, the continuous lines in Fig 4 are based on the values calculated with the pseudo-second-order model. Hubbe et al. [40] reported that the kinetic data for the removal of metals ions and dyes from a wide variety of cellulosic materials (including the biosorbent of the present study) fits this model.

At an initial dye concentration of 15 mg L^-1^, *k*_*2*_ = 1.685 g mg^-1^ h^-1^ and the biosorption equilibrium was reached in 0.25 h. At 300 mg L^-1^, *k*_*2*_ = 0.059 g mg^-1^ h^-1^ and equilibrium was reached in 4 h. According to these results, a higher initial dye concentration gave rise to a lower *k*_*2*_ value and a longer time to reach equilibrium. The value of *k*_*2*_ plays an important role in scaling [41].

### Biosorption isotherm

For each initial dye concentration tested, the equilibrium biosorption capacity (*q*_*eB*_) was related to the residual dye concentration in the solution (*C*_*e*_). The respective data were fitted to two- and three-parameter isotherm models (Table 6), applying non-linear regression analysis on GraphPad Prism^®^ software (version 7.0). The most suitable mathematical model was selected by comparing experimental and theoretical values, based on the error functions (*R*^2^, *SSE* and *RMSE*) obtained. The use of isotherm models helps to explain surface properties of the biosorbent and sorbate as well as the affinity between the two. Some models can estimate maximum biosorption capacity [42, 43].

**Table 6:** Isotherm models.

Model	Equation	Parameters
**Langmuir**	$q_{eB}=\frac{q_{m\;}b_{L}C_{e}}{1\;+\;b_{L}C_{e}}$	*q*_*m*_—Maximum biosorption capacity [mg g^-1^]
		*b*_*L*_—Langmuir constant [L mg^-1^]
**Freundlich**	$q_{eB}\;{=K}_{f}{\;C_{e}}^{1/n_{F}}$	*k*_*F*_—Freundlich constant [mg g^-1^(L/g) ^1/n^]
		*n*_*F*_—Model exponent
**Temkin**	$q_{eB}=\frac{RT}{b_{T}}\text{ln}\;a_{T\;}C_{e}$	*R*—Universal gas constant [8.314 J mol^-1^ K^-1^]
		*T*—Absolute temperature [K]
		*α*_*T*_—Temkin constant [L mg^-1^]
		*b*_*T*_—Temkin constant [J mol^-1^]
**Halsey**	$q_{eB}={\left(\frac{k_{H}}{C_{e}}\right)}^{n_{H}}$	*k*_*H*_—Halsey constant [L g^-1^]
		*n*_*H*_—Model exponent
**Dubinin Radushkevich**	*q*_*eB*_ = *q*_*m*_ exp(−*B*_*D*_*ε*_*D*_^2^)	*q*_*m*_—Maximum biosorption capacity [mg g^-1^]
		*B*_*D*_—Radushkevich constant
		*ε*_*D*_—Polanyi potential [kJ mol^-1^]
**Sips**	$q_{eB}=\frac{ks\;{C_{e}}^{n_{S}}}{1\;+\;bs\;{C_{e}}^{n_{S}}}$	*k*_*s*_—Sips constant [L g^-1^]
		*bs*—Sips constant [L mmol^-1^ o L mg^-1^]
		*n*_*s*_—Model exponent
**Redlich-Peterson**	$q_{eB}=\frac{k_{R}C_{e}}{1\;+\;a_{R}{C_{e}}^{b_{R}}}$	*k*_*R*_—Model constant [L g^-1^]
		*a*_*R*_—Model constant [(L mg^-1^)^-bR^]
		*b*_*R*_—Model exponent
**Radke- Prausnitz**	$q_{eB}=\frac{\alpha_{R}r_{R}{C_{e}}^{\beta_{R}}}{\alpha_{R}\;+\;r_{R}{C_{e}}^{\beta_{R}-1}}$	*α*_*R*_—Model constant [L g^-1^]
		*r*_*R*_—Model constant [L mg^-1^]
		*β*_*R*_—Model exponent
**Toth**	$q_{eB}=\frac{q_{m\;}b_{T}C_{e}}{{\left[1+{{{(b}_{T}C}_{e})}^{1/n_{T}\;}\;\right]}^{n_{T}}}$	*q*_*m*_—Maximum biosorption capacity (mg g^-1^)
		*b*_*T*_—Affinity parameter
		*n*_*T*_—Heterogeneity parameter

**Fig 5:**
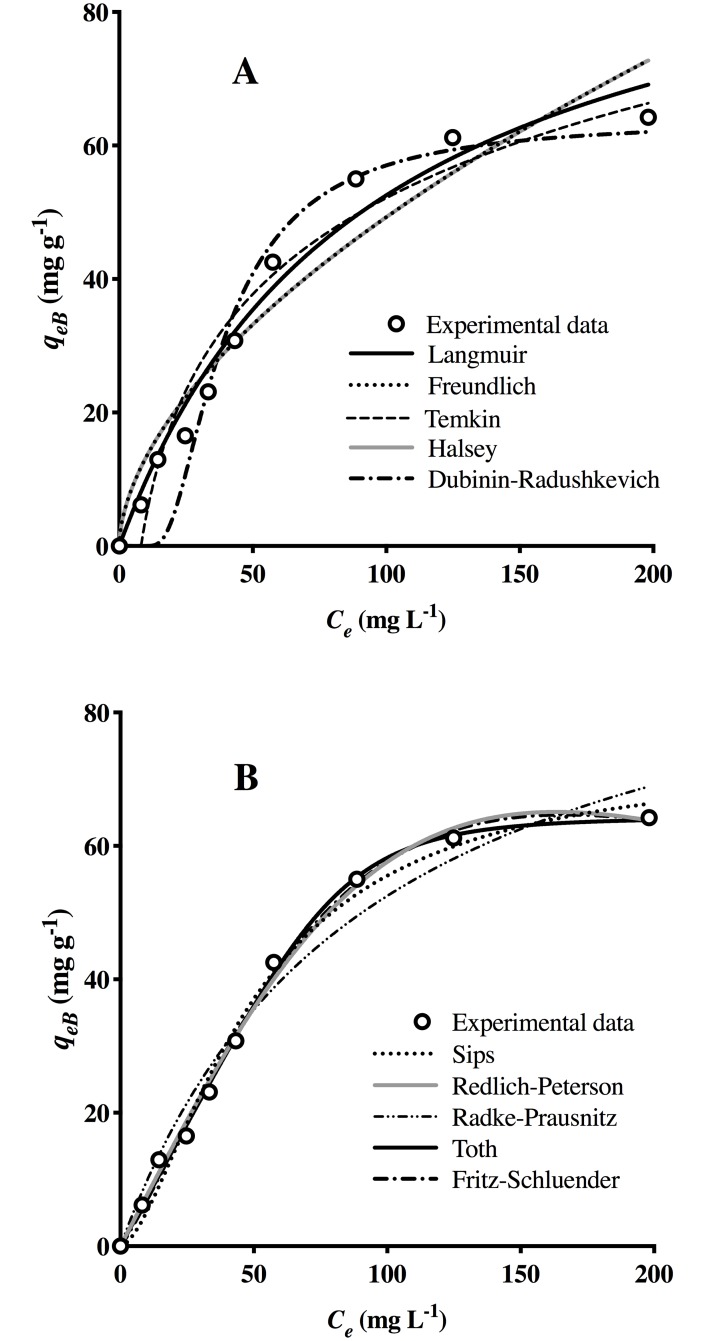
Two- (A) and three-parameter (B) isotherms for the biosorption of AO74 by HPL.

Fig 5 shows the experimental *q*_*eB*_ versus *C*_*e*_ and the behavior predicted by the two- (A) and three-parameter (B) biosorption isotherm models.

The isotherm parameters predicted by the models chosen to analyze the data are listed in Table 7, as are the corresponding error functions (*R*^*2*^, *SSE* and *RMSE*).

**Table 7:** Isotherm model parameters for the biosorption of AO74 by HPL.

Two-parameter models		Three-parameter models	
**Langmuir**		**Sips**	
***b* (L mg**^**-1**^**)**	0.011 ± 0.002	***k***_***s***_ **(L g**^**-1**^**)**	0.002 ± 0.001
***q***_***m***_ **(mg g**^**-1**^**)**	101.9 ± 10.82	***q***_***m***_ **(mg g**^**-1**^**)**	73.53 ± 5.104
**R**^**2**^	0.976	***n***_***S***_	1.602 ± 0.202
**SSE**	119.5	**R**^**2**^	0.991
**RMSE**	3.864	**SSE**	46.13
		**RMSE**	2.567
**Freundlich**		**Redlich-Peterson**	
***k***_***F***_ **(mg g**^**-1**^ **(L g**^**-1**^**)**^**1/n**^**)**	3.57 ± 1.212	***k***_***R***_ **(L g**^**-1**^**)**	0.771 ± 0.0361
***n***_***F***_	1.754 ± 0.223	***a***_***R***_ **(L mg**^**-1**^**)**^**-BR**^	0.00002 ± 0.00003
**R**^**2**^	0.937	***B***_***R***_	2.068 ± 0.240
**SSE**	311.3	**R**^**2**^	0.996
**RMSE**	6.238	**SSE**	21.24
		**RMSE**	1.742
**Temkin**		**Radke-Prausnitz**	
***a***_***T***_ **(L mg**^**-1**^**)**	0.123 ± 0.021	***a***_***R***_ **(L g**^**-1**^**)**	1.092 ± 0.356
***b***_***T***_ **(J mol**^**-1**^**)**	51.81 ± 4.495	***r***_***R***_ **(L mg**^**-1**^**)**	101.1 ± 205.9
**R**^**2**^	0.950	***B***_***R***_	2.21E-08 ± 0.366
**SSE**	191	**R**^**2**^	0.976
**RMSE**	5.223	**SSE**	119.6
		**RMSE**	4.133
**Halsey**		**Toth**	
***k***_***H***_ **(L g**^**-1**^**)**	0.1073 ± 0.101	***q***_***m***_ **(mg g**^**-1**^**)**	64.23 ± 1.527
***n***_***H***_	-0.57 ± 0.078	***b***_***T***_ **(L mg**^**-1**^**)**	0.011 ± 0.0004
**R**^**2**^	0.918	**n**_**T**_	0.223 ± 0.050
**SSE**	311.3	**R**^**2**^	0.997
**RMSE**	6.668	**SSE**	13.25
		**RMSE**	1.376
**Dubinin-Radushkevich**			
***q***_***m***_ **(mg g**^**-1**^**)**	63.84 ± 4.144		
***B***_***D***_ **(mol**^**2**^ **KJ**^**-2**^**)**	0.001 ± 0.0002		
**R**^**2**^	0.930		
**SSE**	266.6		
**RMSE**	6.172		

When comparing error functions (Table 7), it becomes apparent that the correlation coefficients of three-parameter models are generally higher than those of two-parameter models. The Freundlich, Temkin and Halsey models did not approximate the experimental isotherm. The two-parameter models showing the best description of the experimental data were Langmuir and Dubinin-Radushkevich. However, the maximum biosorption capacity predicted by Langmuir (101.9 mg g^-1^) was much greater than the experimental value (64.24 mg g^-1^). Although the Dubinin-Radushkevich isotherm predicted an equilibrium biosorption capacity close to the experimental value (63.84 mg g^-1^ versus 64.24 mg g^-1^), the estimated *R*^*2*^ value was low (0.93). Hence, these models were not apt for calculating the equilibrium of AO74 biosorption by HPL.

Of the three-parameter models, the Toth isotherm best represents the experimental results (*q*_*m*_ = 64.23 mg g^-1^, *R*^*2*^ = 0.997, *SSE* = 13.25, *RSME* = 1.376). The value of *n*_*T*_ (0.223) indicates the heterogeneous character of the biosorption system under study. This model was selected as the best for depicting the AO74 biosorption equilibrium achieved by HPL. The Toth isotherm is a useful empirical equation for describing heterogeneous adsorption systems at low and high sorbate concentrations. It assumes a quasi-Gaussian energy distribution, implying that the most efficient binding sites have a sorption energy below the mean value [44]. McKay et al. [45] demonstrated the suitability of the Toth isotherm for estimating the experimental equilibrium sorption of Acid Yellow 117 (AY117), Acid Red 114 (AR114) and Acid Blue 80 (AB80) on activated carbon. Likewise, Han et al. [46] identified the same isotherm model as the most appropriate for biosorption of Neutral Red by peanut shells.

Regarding the biosorption of AO74 by traditional sorbents, no previous report exists, to our knowledge, that could serve as a basis of comparison for the current data on the maximum experimental removal capacity or the values of the parameters indicating the best model for predicting biosorption equilibrium. Only two descriptions were found in the literature related to the removal of AO74. Sharma and Dutta [5] studied the photocatalytic degradation of this dye and Gurbuz et al. [47] treated an AO74-polluted effluent with a bio-composite, employing *Aspergillus niger* for the decolorization of the dye.

### Desorption studies

An examination of dye desorption provides insights into the biosorption process and mechanism. The adequate selection of an eluent solution allows for biosorbent regeneration and makes the treatment process more economical and feasible [48]. The recovery of a sorbate enables it to be reused, thus avoiding disposal in the environment and the consequent damage to ecosystems. Hence, desorption should be considered as an important step in the design of a bioremediation strategy.

**Fig 6:**
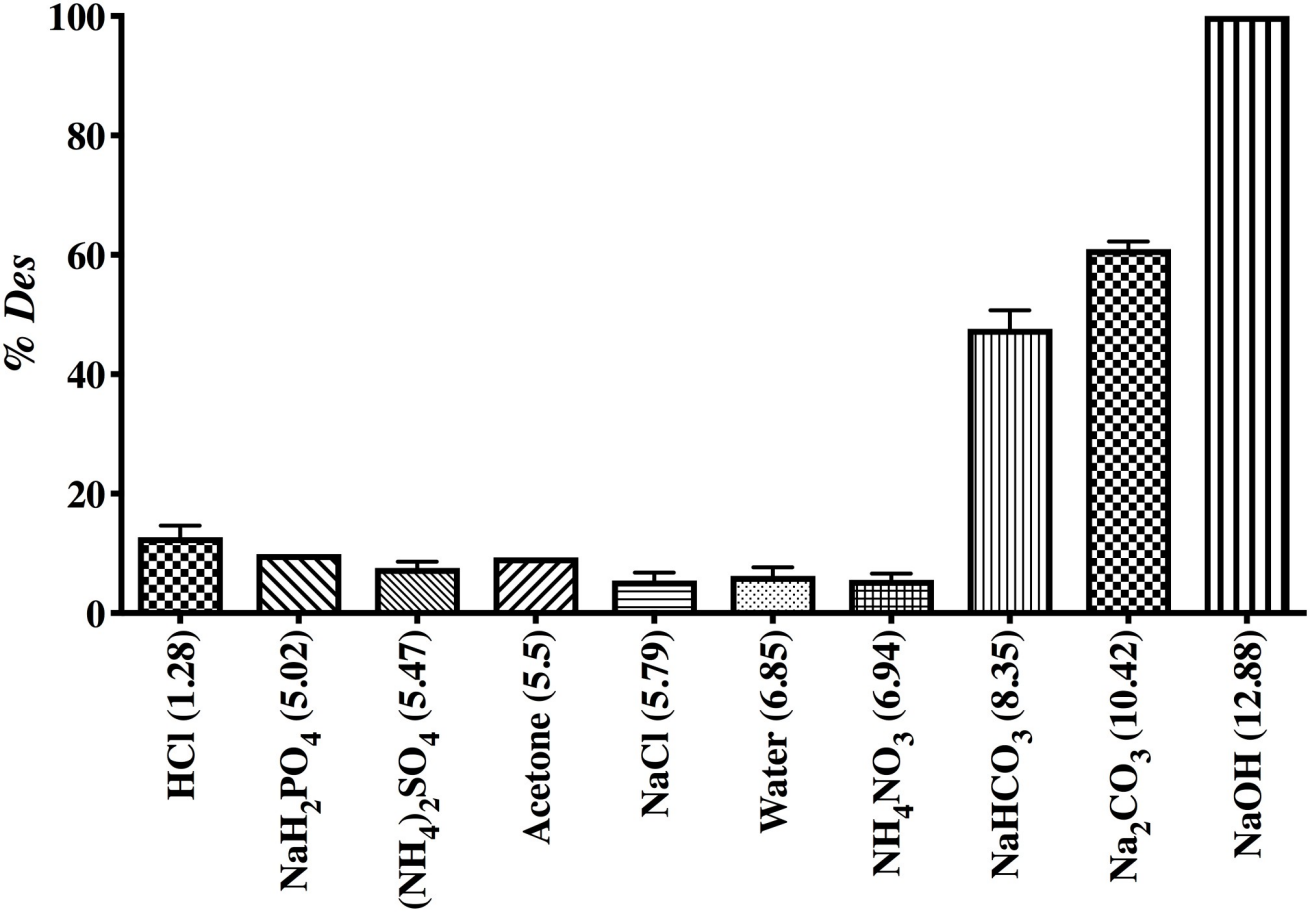
Percentage of desorption promoted by different eluent solutions.

Fig 6 shows the ten eluent solutions used to test the desorption of AO74 from AO74-loaded HPL as well as their corresponding pH values. The percentage of dye desorption (% *Des*) attained with distilled deionized water was very low (12.7%). If water had completely eluted the dye, the binding of the sorbent to the sorbate would have been accomplished through weak bonds [49]. Alkaline solutions (pH >8) promoted the greatest percentage of desorption: NaOH (100%), Na_2_CO_3_ (60%) and NaHCO_3_ (50%).

The pH of the solutions could be a pivotal factor in explaining these results. At higher pH values there was a greater negative charge of the biosorbent surface, according to the determination of the zeta potential. Hence, alkaline solutions may boost electrostatic repulsion between the biosorbent and the anionic dye, leading to the elution of AO74. Solutions having a pH close to neutral or in the acidic range induced a maximum of 12% dye recovery. The present findings concur with a previous report describing the enhancement of anionic dye sorption under acidic conditions and of desorption under alkaline conditions [10].

**Fig 7:**
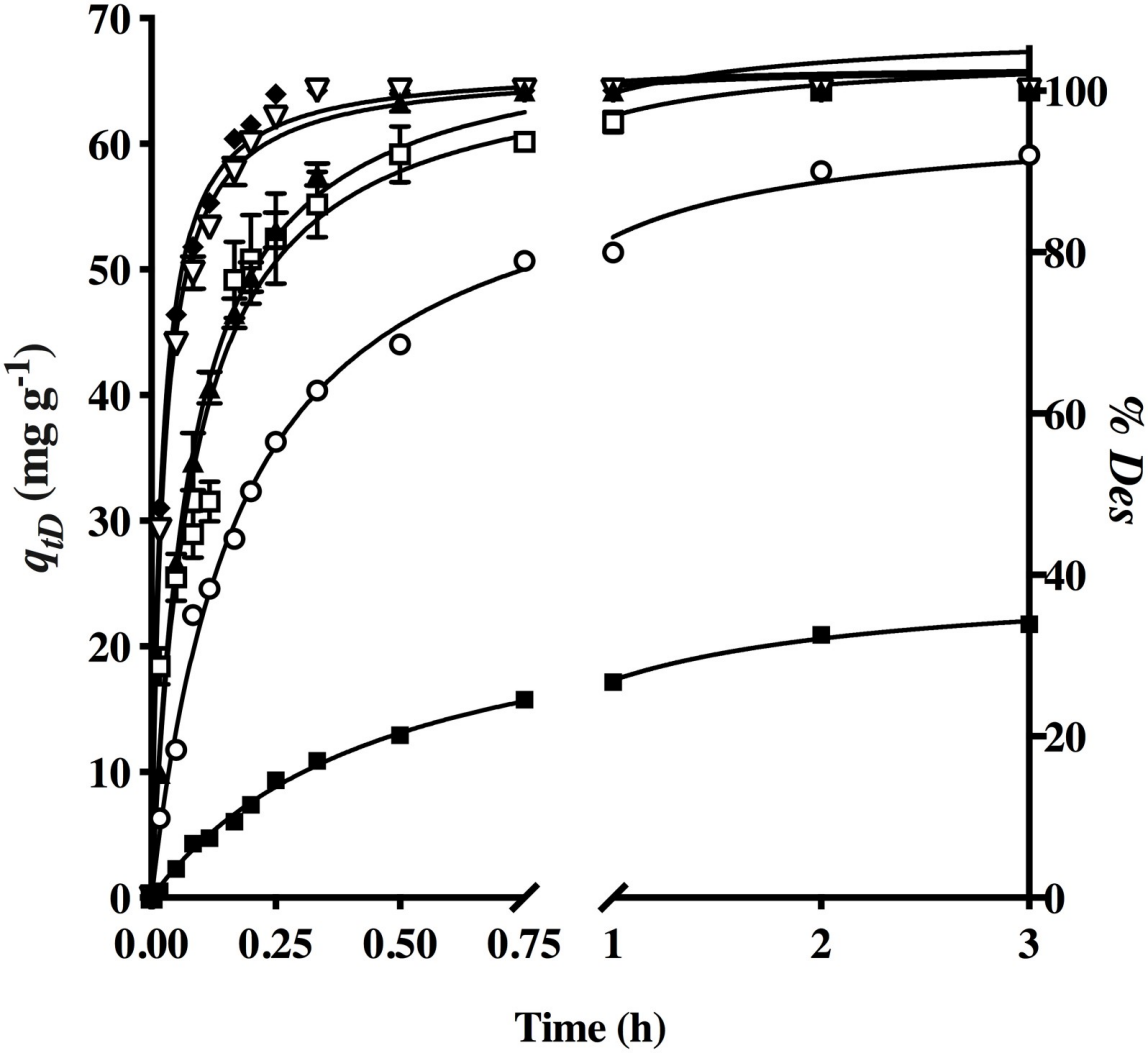
Effect of different concentrations of NaOH on dye desorption from AO74-loaded HPL [NaOH concentrations (M) at (■) 0.001, (○) 0.005, (▲) 0.01, (▽) 0.05, (◆) 0.1 and (□) 0.5] and (—) the pseudo-second-order model prediction.

Accordingly, it was decided to examine the kinetics of desorption by utilizing various concentrations of NaOH as the eluent solution (Fig 7). At the lowest concentrations (0.001 and 0.005 M), it was not possible to desorb 100% of the dye from AO74-loaded HPL. At all other concentrations, the dye was completely desorbed (64.24 mg g^-1^). Within the range of 0.01 to 0.1 M, an increment in the concentration of NaOH resulted in a shorter time (from 0.5 to 0.2 h) to accomplish 100% desorption of the dye and reach desorption equilibrium (*t*_*eD*_). At a higher concentration of NaOH (0.5 M), contrarily, the time to reach *t*_*eD*_ increased to 0.75 h (Table 8).

**Table 8:** Kinetic model parameters for dye desorption from AO74-loaded HPL at different concentrations of NaOH.

NaOH (M)	0.001	0.005	0.01	0.05	0.1	0.5
***t***_***eD***_ **(h)**	2.0	2.0	0.5	0.25	0.2	0.75
***q***_***eD***_ ***exp* (mg g**^**-1**^**)**	21.35 ± 0.42	58.47 ± 0.64	64.05 ± 0.19	63.93 ± 0.31	63.86 ± 0.34	62.59 ± 1.00
**Pseudo-first-order model**						
***q***_***e1***_ **(mg g**^**-1**^**)**	21.03 ± 0.23	54.57 ± 0.75	63.28 ± 0.47	62.37 ± 0.58	62.74 ± 0.50	61.66 ± 0.96
***k***_***1***_ **(h**^**-1**^**)**	2.05 ± 0.06	4.49 ± 0.18	8.63 ± 0.23	24.27 ± 1.36	28.25 ± 1.47	8.25 ± 0.46
**R**^**2**^	0.991	0.976	0.989	0.964	0.971	0.951
**SSE**	23.1	443.4	256.8	637.3	510	1033
**RMSE**	0.6541	2.865	2.181	3.435	3.073	4.374
**Pseudo-second-order model**						
***q***_***e2***_ **(mg g**^**-1**^**)**	25.49 ± 0.22	62.14 ± 0.45	69.08 ± 0.54	66.09 ± 0.30	66.22 ± 0.28	67.36 ± 1.15
***k***_***2***_ **(g mg**^**-1**^ **h**^**-1**^**)**	0.083 ± 0.002	0.089 ± 0.002	0.184 ± 0.007	0.649 ± 0.023	0.763 ± 0.027	0.180 ± 0.016
**R**^**2**^	0.997	0.996	0.992	0.994	0.994	0.959
**SSE**	7.829	80.37	196.3	108.2	97.36	866.9
**RMSE**	0.3808	1.22	1.908	1.416	1.343	4.007
**Elovich model**						
***A* (mg g**^**-1**^ **h**^**-1**^**)**	155.9 ± 8.371	977.9 ± 52.18	3555 ± 703.1	197146 ± 127956	537784 ± 447882	4024 ± 887.2
***B* (g mg**^**-1**^**)**	0.215 ± 0.006	0.088 ± 0.002	0.091 ± 0.005	0.159 ± 0.012	0.175 ± 0.016	0.098 ± 0.005
**R**^**2**^	0.960	0.980	0.879	0.761	0.712	0.865
**SSE**	92.46	282.8	1716	1298	1376	1689
**RMSE**	1.36	2.378	5.858	5.095	5.245	5.812
**Fractional power model**						
***k***_***p***_ **(mg g**^**-1**^**)**	15.31 ± 0.28	48.42 ± 0.89	61.14 ± 1.48	64.37 ± 1.08	64.79 ± 1.08	59.41 ± 1.32
***v* (h**^**-1**^**)**	0.421 ± 0.017	0.287 ± 0.015	0.193 ± 0.017	0.10 ± 0.010	0.089 ± 0.010	0.193 ± 0.016
**R**^**2**^	0.943	0.906	0.763	0.687	0.644	0.785
**SSE**	133.9	1271	3361	1696	1703	2693
**RMSE**	1.636	5.042	8.199	5.824	5.837	7.339

Zhang and Wang [39] studied the desorption of nickel (II) from a nanocomposite (lignocellulose/montmorillonite). They observed enhanced desorption capacity with an greater concentration of HNO_3_, attributed to the fact that a rise in the concentration of H^+^ ions in the solution increased the concentration gradient between Ni^2+^ and H^+^ and consequently intensified the driving force of the desorption process by an ion-exchange mechanism. At very high acid concentrations, on the other hand, an excessive rise in the concentration of H^+^ ions may have exacerbated electrostatic repulsion with the nickel ions and therefore inhibited desorption.

Among the various theoretical models presently employed for calculating the kinetic parameters of desorption (Table 8), the pseudo-second-order model best fitted experimental data (continuous lines in Fig 7), as was found for biosorption as well.

According to the results, a concentration of 0.01 M NaOH was able to completely remove the dye from AO74-loaded HPL in a short time, thus maintaining the structural integrity of the biosorbent. Hence, this hydroxide concentration was utilized in the rest of the assays.

### Biosorption and desorption cycles

**Fig 8:**
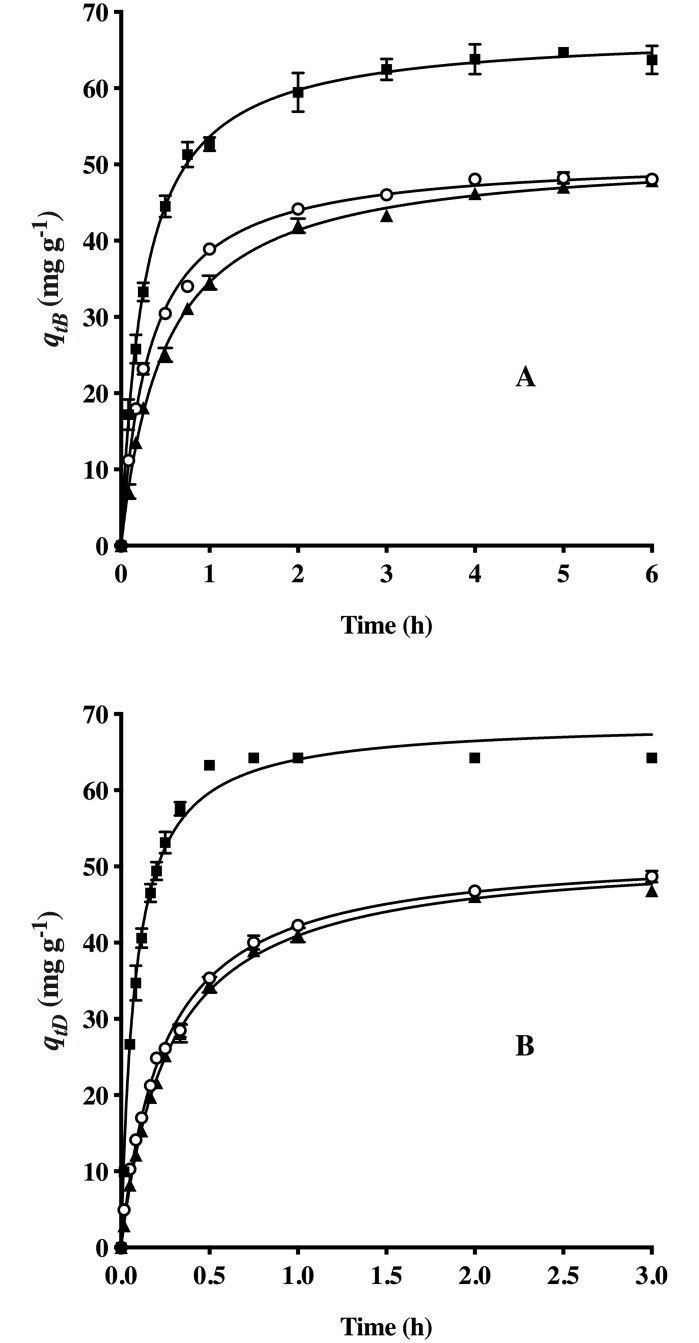
Kinetics of dye biosorption by HPL (A) and desorption from AO74-loaded HPL (B) during three cycles [(■) 1, (○) 2 and (▲) 3] and (—) the pseudo-second-order model prediction.

The biosorbent was examined during three biosorption and desorption cycles to analyze possible changes in the corresponding capacities of HPL and NaOH (Fig 8).

The biosorption equilibrium capacity of the first cycle was 64.24 mg g^-1^. Although this parameter diminished considerably (25.1%) in the second cycle to 48.11 mg g^-1^, from the second to the third cycle there was no significant change (*q*_*eB*_ = 47.5 mg g^-1^ approx.). The dye removal rate decreased over the three cycles, reflected in the *k*_*2*_ values of the pseudo-second-order model (Table 9A), which best described the experimental kinetic data.

**Table 9:** Kinetic model parameters for the biosorption (A) and desorption (B) of AO74 over three cycles.

	(A) Biosorption			(B) Desorption		
Cycle number	1	2	3	1	2	3
***t***_***e***_ **(h)**	4.0	4.0	4.0	0.5	2.0	2.0
***q***_***e***_ ***exp* (mg g**^**-1**^**)**	64.24 ± 0.20	48.11 ± 0.056	47.04 ± 0.49	64.05 ± 0.19	47.73 ± 0.95	46.46 ± 0.39
**Pseudo-first-order model**				**Pseudo-first-order model**		
***q***_***e1***_ **(mg g**^**-1**^**)**	62.49 ± 0.49	46.31 ± 0.44	45.54 ± 0.37	63.28 ± 0.47	45.34 ± 0.63	44.73 ± 0.43
***k***_***1***_ **(h**^**-1**^**)**	2.71 ± 0.096	2.285 ± 0.084	1.634 ± 0.047	8.63 ± 0.23	3.547 ± 0.136	3.223 ± 0.084
**R**^**2**^	0.975	0.9766	0.9865	0.989	0.979	0.991
**SSE**	859.5	407.9	249.7	256.8	262.9	115.7
**RMSE**	3.238	2.414	1.889	2.181	2.206	1.464
**Pseudo-second-order model**				**Pseudo-second-order model**		
***q***_***e2***_ **(mg g**^**-1**^**)**	66.88 ± 0.34	51.0 ± 0.20	51.57 ± 0.22	69.08 ± 0.54	52.22 ± 0.40	52.02 ± 0.29
***k***_***2***_ **(g mg**^**-1**^ **h**^**-1**^**)**	0.059 ± 0.002	0.061 ± 0.001	0.039 ± 0.001	0.184 ± 0.007	0.080 ± 0.002	0.071 ± 0.002
**R**^**2**^	0.992	0.9974	0.9978	0.992	0.996	0.998
**SSE**	278.3	44.57	41.42	196.3	51.72	23.97
**RMSE**	1.842	0.7979	0.7693	1.908	0.979	0.666
**Elovich model**				**Elovich model**		
***A* (mg g**^**-1**^ **h**^**-1**^**)**	1650 ± 354.1	470.8 ± 30.83	260.5 ± 9.83	3555 ± 703.1	638.7 ± 33.08	523 ± 26.52
***B* (g mg**^**-1**^**)**	0.109 ± 0.005	0.111 ± 0.002	0.101 ± 0.001	0.091 ± 0.005	0.1053 ± 0.002	0.1028 ± 0.002
**R**^**2**^	0.870	0.9749	0.9872	0.879	0.977	0.974
**SSE**	2531	264.5	162.1	1716	222	260.2
**RMSE**	5.771	2.033	1.592	5.858	2.107	2.281
**Fractional power model**				**Fractional power model**		
***k***_***p***_ **(mg g**^**-1**^**)**	46.98 ± 0.980	33.69 ± 0.51	29.96 ± 0.53	61.14 ± 1.48	38.31 ± 0.65	36.85 ± 0.74
***v* (h**^**-1**^**)**	0.160 ± 0.011	0.250 ± 0.011	0.3077 ± 0.013	0.193 ± 0.017	0.318 ± 0.014	0.338 ± 0.017
**R**^**2**^	0.7696	0.9156	0.9298	0.763	0.928	0.9119
**SSE**	4497	889.4	892.2	3361	691.6	889.7
**RMSE**	7.692	3.728	3.734	8.199	3.719	4.218

Although desorption with 0.01 M NaOH was efficient during all three cycles (achieving 100% removal of the dye from the AO74-loaded biosorbent), a significant difference was detected in the desorption capacity between the first and second cycles. The kinetic behavior was similar during the second and third cycles, but evidently slower in the latter, as revealed by the lower *k*_*2*_ value (Table 9B). This finding suggests a deterioration of the biosorbent. It is likely that the extreme pH conditions involved in removing (pH 2) and subsequently eluting (pH 12) AO74 caused a loss of some biosorption sites.

### FTIR analysis

**Fig 9:**
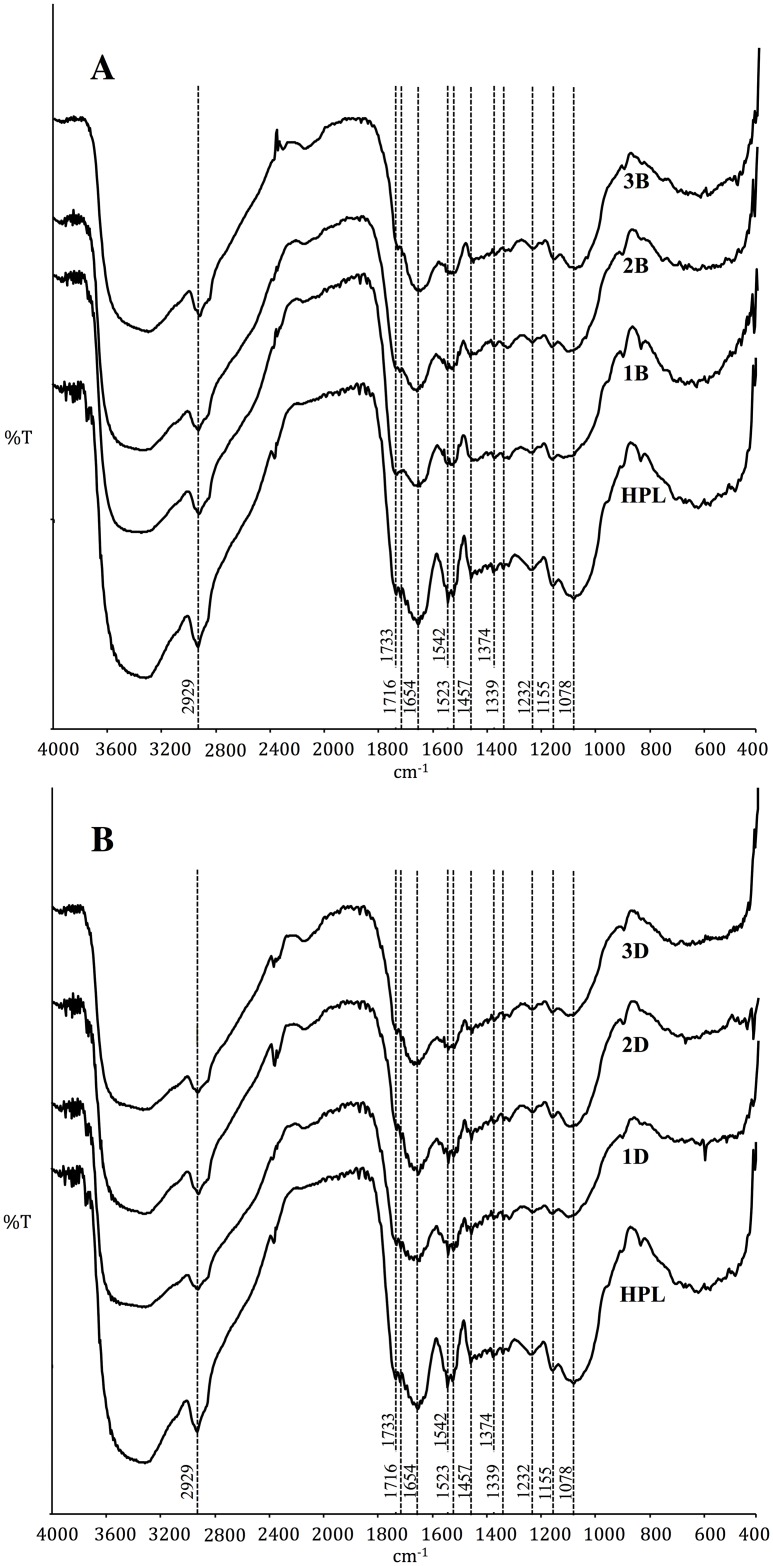
FTIR spectra of HPL, dye-loaded HPL (A) and dye-desorbed HPL (B) during three cycles.

The FTIR spectrum of HPL (Fig 9A) displayed a broad band at 3900–3300 cm^-1^ due to the stretching vibration of -OH and −NH groups. The absortion peak at 2929 cm^-1^ was assigned to the C-H group [25]. Among the other frequencies, 1733 and 1716 cm^-1^ were assigned to the stretching of the -COO- group [20], 1654 cm^-1^ to the C = O group of amide I, 1542 and 1523 cm^-1^ to the C-N stretching and N-H bending of amide II produced by the proteins in the biosorbent [25, 50], and 1457 cm^-1^ to the phenolic O-H vibration [20] and C-O stretching of the carboxylates [24]. Furthermore, 1374 cm^-1^ was attributed to C-N stretching, O-H bending and the presence of sulfur- and phosphorus-containing compounds [51]. The 1339 cm^-1^ frequency was ascribed to the C-H vibration in cellulose [50] or the N-H bond of the amine group [52], 1232 cm^-1^ to C-O stretching (suggesting the presence of hemicellulose) [35], and 1155 and 1078 cm^-1^ to the C-O-C and -OH vibration of polysaccharides [20, 50, 53]. The latter bands can also be assigned to the Si-O bond and to the phosphate groups commonly occurring in aquatic biomass [37].

FTIR spectra of HPL and dye-loaded HPL for the three cycles (Fig 9A, 1B-3B) showed an absence of bands at 1733, 1716, 1542 and 1339 cm^-1^ following AO74 biosorption. These peaks are characteristic of carboxyl and amide groups, which evidently playing an important role in dye uptake. In another study, the carboxyl and amide groups were identified as the biosorption sites for arsenic ions on the cell wall of *Lemna minor* [53]. In the current contribution, the vibration frequency at 1155–1078 cm^-1^ decreased after AO74 biosorption, probably caused by a modification in the polysaccharide structure of the plant cell wall or the removal of the Si-O and/or phosphate group (the latter prompted by the acidic pH used for biosorption).

A similarity should be found between the FTIR spectra of HPL at the beginning of the first cycle and AO74-desorbed HPL at the end of each cycle (Fig 9B, 1D-3D). Subsequent to the first dye elution, the reappearance of the peaks at 1734 and 1716 cm^-1^ indicates an apparent recovery of the carboxyl groups, although they were weaker than in the original HPL spectrum. Moreover, there was a remarkable reduction in the intensity of the bands ranging from 1654 to 1457 cm^-1^ and from 1232 to 1078 cm^-1^, the expected frequencies for the amide and −OH groups as well as the polysaccharide-C-O-C bond.

These changes may be due to the effect of the eluent solution (NaOH) on proteins and some soluble polysaccharides [54]. The results suggest a likely decline in the number of active biosorption sites following the first cycle, which would explain the lower biosorption capacity during the second and third cycles. Additionally, a comparison of fingerprint regions (800 and 400 cm^-1^) of the biosorbent at the end of each of the three cycles demonstrates the existence of differences in its structure.

The FTIR analysis corroborated the relevance of the amide groups in the removal of AO74 by HPL. This is consistent with the fact that the metal-complex acid dyes are good for coloring materials containing nitrogen, such as polyamide fibers, silk, nylon, wool and leather [1, 55].

### SEM/EDX analysis

**Fig 10:**
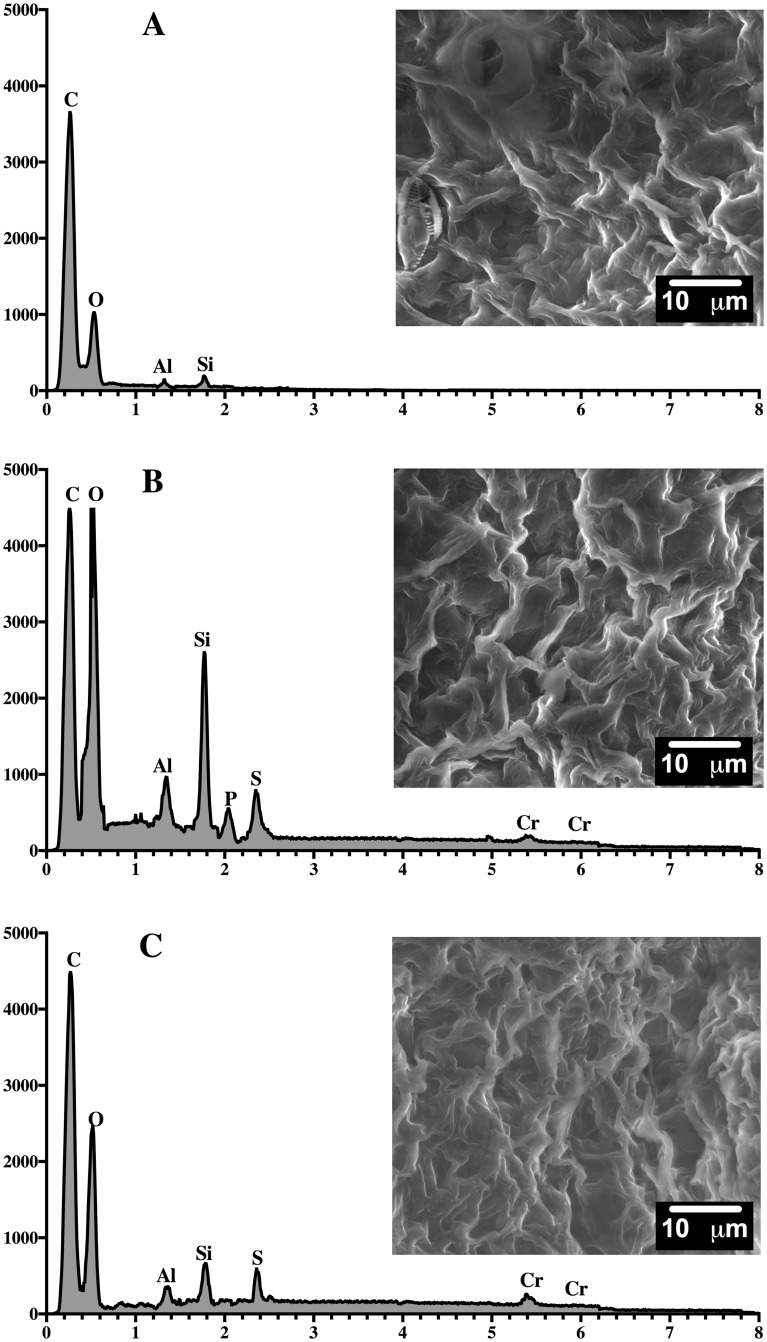
SEM micrographs (2500x)/EDX spectra of HPL (A) and AO74-loaded HPL after the first (B) and third (C) steps of biosorption.

**Fig 11:**
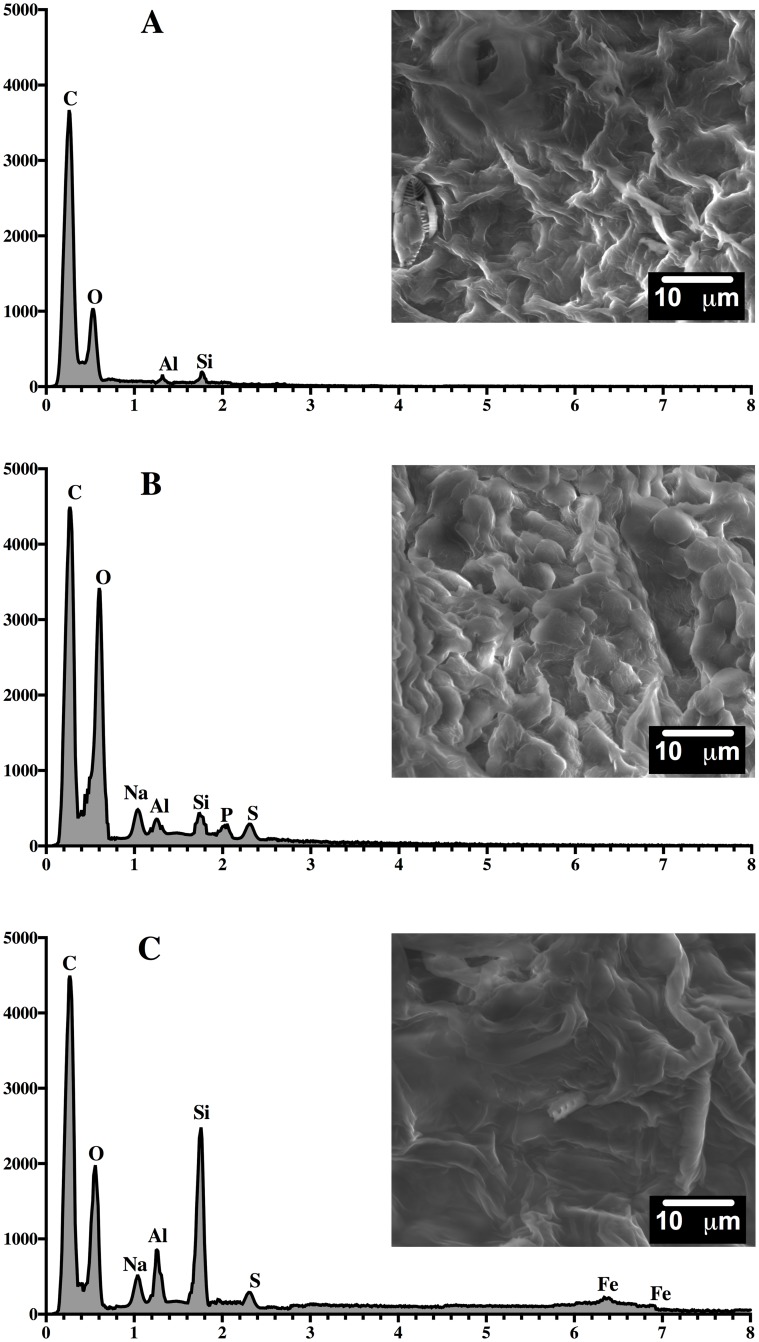
SEM micrographs (2500x)/EDX spectra of HPL (A) and AO74-desorbed HPL after the first (B) and third (C) steps of desorption.

The SEM/EDX analysis was carried out on the biosorbent (HPL) as well as dye-loaded HPL and dye-unloaded HPL at the end of the first and third steps of biosorption (Fig 10) and desorption (Fig 11).

The SEM/EDX images of HPL (Figs 10A and 11A) show a net-like biomaterial with a large number of deep pores irregular in shape and size. No chromium was detected on the HPL surface. No significant structural modifications were found in HPL subsequent to the first biosorption step (Fig 10B). The pores seem to have lost depth and the surface appears smoother than in dye-unloaded HPL, possibly due to the interaction between the dye and the biosorption sites. Nevertheless, after the third step of biosorption (Fig 10C), a dense surface with smoother pores emerged, indicating a structural alteration in the biosorbent.

Upon completing the first and third steps of desorption, the biosorbent had not completely recovered its original structure (Fig 11B and 11C). Although HPL regained its net-like shape in some areas, conglomerates can be seen in other areas, causing a decline in the number of pores and available contact surface for dye removal. These structural changes in the biosorbent revealed by SEM micrographs and FTIR spectra may explain the reduced capacity of HPL for the biosorption of AO74 during the second and third cycles.

EDX analysis corroborated the presence of chromium at the point of biosorbent saturation (Fig 10B and 10C). The chromium ion constitutes the heavy metal in AO74, being responsible for its high toxicity and persistence in the environment. This observation corroborates dye biosorption by HPL. EDX spectra of the eluted biosorbent (Fig 11B and 11C) demonstrated the absence of chromium, verifying that the 0.01 M NaOH solution completely removed the dye from the HPL surface, as indicated by desorption kinetics. According to the SEM/EDX results, the acidic conditions for dye removal followed by alkaline treatment for its elution could possibly be aggressive to HPL, thus modifying its structure and affecting its biosorption capacity for subsequent cycles.

### CLSM analysis

Confocal microscopy is based on the fluorescent property of some compounds. Components in these compounds absorb and emit light at certain wavelengths, producing what is known as primary fluorescence or autofluorescence [56]. In the current contribution, CLSM was performed to verify the presence of the AO74 dye on the HPL structure after the biosorption process. The micrograph for HPL displayed a peak characteristic of fluorescence emission signaling at 676 nm, while that for AO74 exhibited such characteristic peaks at 539, 576 and 676 nm. Chlorophyll, carotenes and xanthophylls are known to absorb and emit light in plants, and therefore are considered the source of their primary fluorescence [56, 57]. The autofluorescence of azo dye molecules, on the other hand, is produced by their aromatic components. Since not all emission signals of HPL and AO74 overlap, the components responsible for generating the signals could be distinguished from one another in the CLSM analysis.

**Fig 12:**
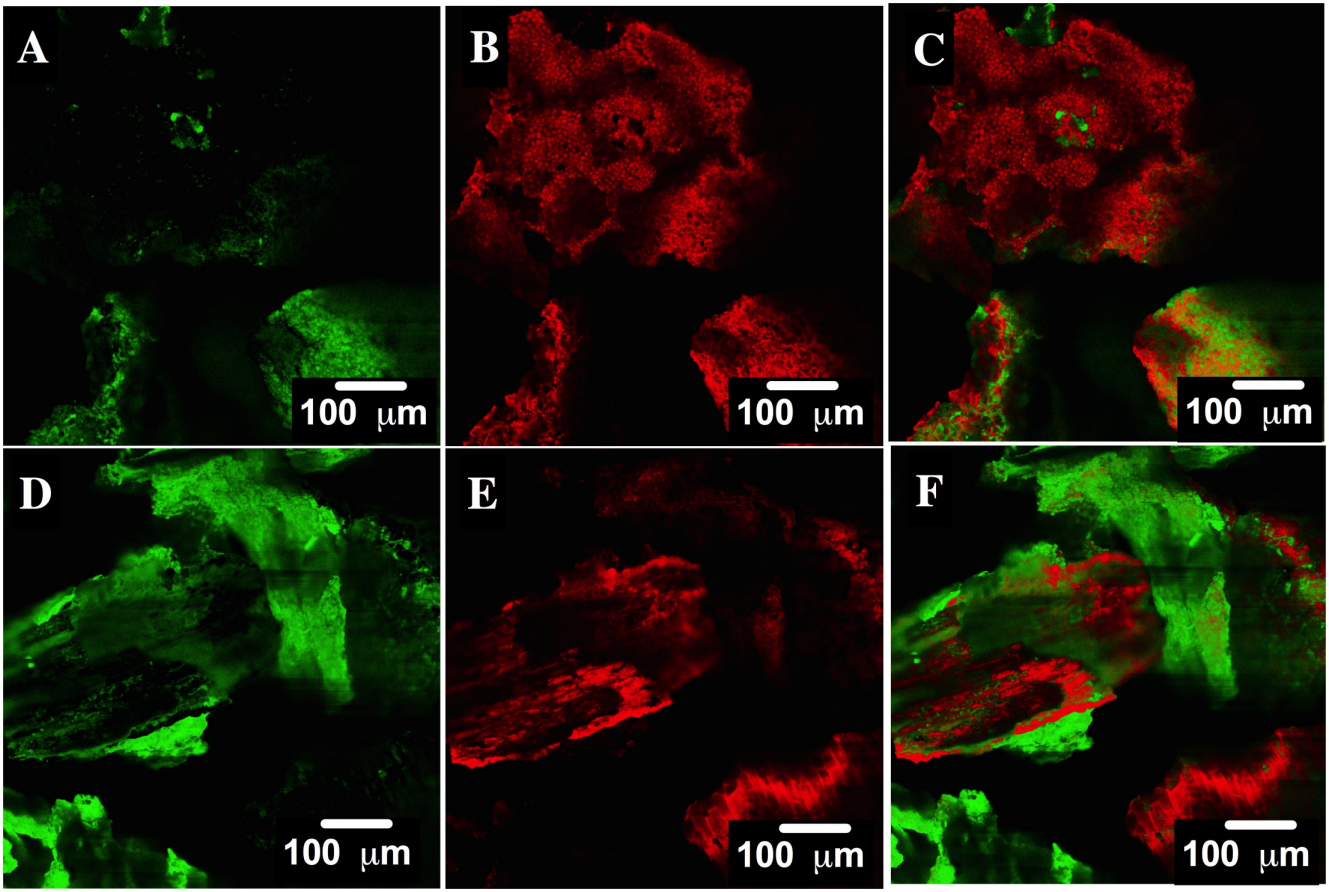
CLSM images of HPL in green, acid orange 74 (AO74) in red, and the overlap of both showing their interaction after the first (A, B and C) and the third step (D, E and F) of biosorption.

**Fig 13:**
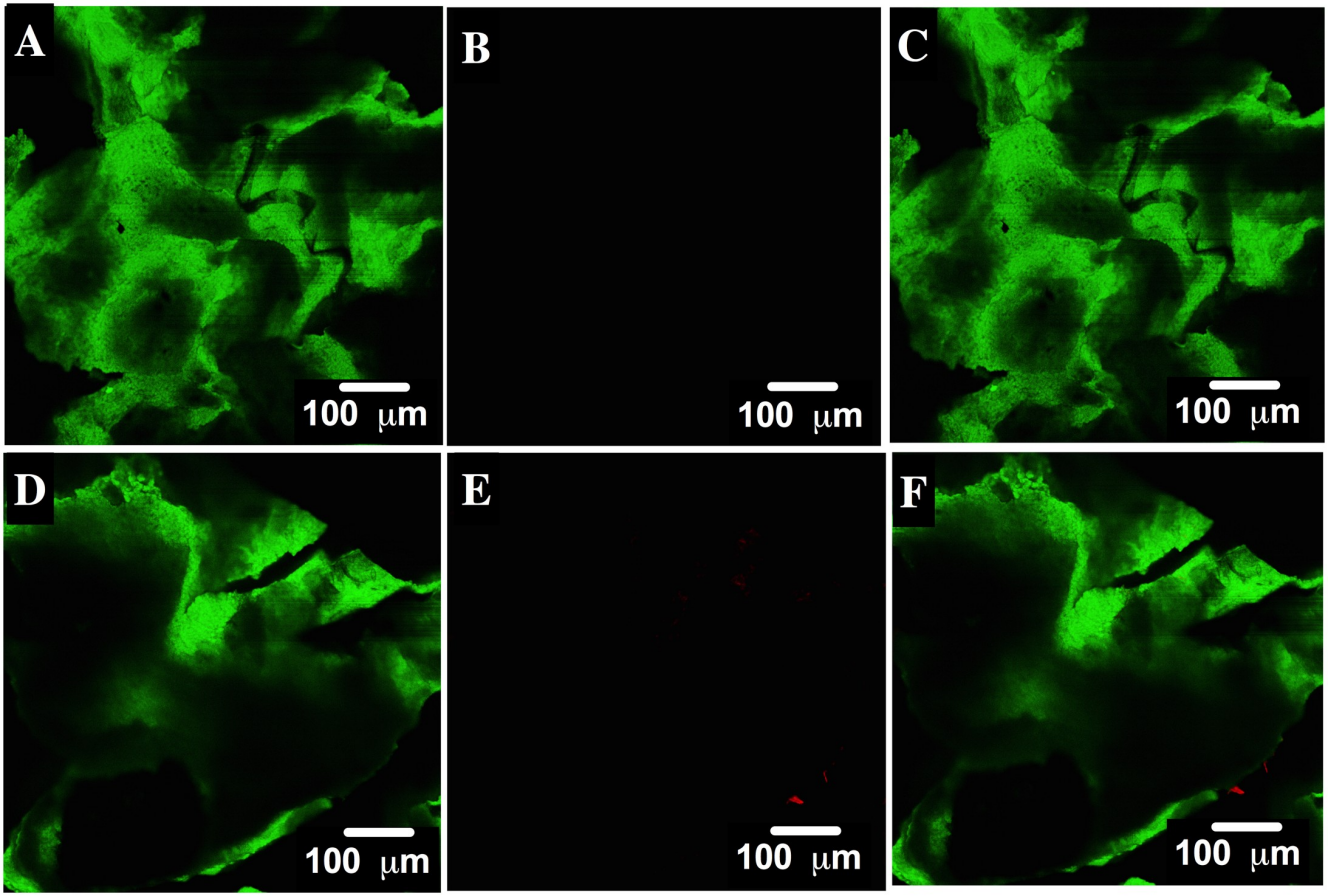
CLSM images of HPL in green, acid orange 74 (AO74) in red, and the overlap of both showing their interaction after the first (A, B and C) and the third step (D, E and F) of desorption.

CLSM images are shown for the first (Fig 12A–12C) and third steps (Fig 12D–12F) of biosorption. Fluorescence is portrayed in green for HPL (Fig 12A and 12D) and in red for AO74 (Fig 12B and 12E). The overlap is also illustrated (Fig 12C and 12F). The micrographs demonstrate that the AO74 dye was on the surface of HPL after biosorption. Contrarily, the CLSM micrographs of the first and third steps of the desorption process show the fluorescence (in green) of HPL (Fig 13A and 13D, respectively) and the absence of the fluorescence of the dye (Fig 13B and 13E, respectively). These results confirm the complete removal of AO74 from the surface of HPL. The current study evidences the utility of the CLSM technique for corroborating the uptake of a sorbate on a biosorbent. To date, few reports exist on the use of this technique to examine the sorption of heavy metals [58, 59, 60] and dyes [61].

## Conclusions

HPL herein exhibited a remarkable capacity (64.24 mg g^-1^) for the biosorption of AO74 from an aqueous solution at pH 2. The pseudo-second-order model was the most suitable for describing the biosorption kinetics of solutions having different pH values and initial concentrations of the dye. The Toth isotherm model was the best for explaining biosorption data at equilibrium, pointing to the heterogeneous nature of the HPL surface.

According to the evaluation of desorption, 0.01 M was an appropriate concentration of NaOH for three effective biosorption-desorption cycles. Although biosorption capacity decreased (64.24> 48.11> 47.04 mg g^-1^), the dye was completely removed from AO74-loaded HPL during each cycle.

FTIR spectroscopy revealed that the carboxyl and amide groups of the proteins of HPL constitute the main biosorption sites for dye removal. Additionally, it showed a loss of HPL proteins and polysaccharides during the three cycles. SEM micrographs displayed a change in the structure of HPL after the third step of AO74 biosorption. Hence, polysaccharides from the plant also play a key role in the biosorption of AO74 by HPL. Both SEM/EDX and CLSM images verified the presence of the dye on the surface of HPL.

The performance of HPL during three biosorption-desorption cycles strongly suggests that HPL is an effective and low-cost biosorbent. Furthermore, the ability to carry out desorption of AO74 from HPL avoids the generation of toxic sludge in the biosorption process.
